# Trauma-informed care for children in the ambulance: international survey among pre-hospital providers

**DOI:** 10.1080/20008198.2016.1273587

**Published:** 2017-02-08

**Authors:** Eva Alisic, Mark P. Tyler, Melita J. Giummarra, Rahim Kassam-Adams, Juul Gouweloos, Markus A. Landolt, Nancy Kassam-Adams

**Affiliations:** ^a^ Monash University Accident Research Centre, Monash University, Clayton, Australia; ^b^ Department of Psychosomatics and Psychiatry, University Children’s Hospital Zurich, Zurich, Switzerland; ^c^ School of Psychological Science and Monash Institute of Cognitive and Clinical Neurosciences, Monash University, Clayton, Australia; ^d^ School of Psychology and Public Health, Department of Psychology and Counselling, La Trobe University, Melbourne, Australia; ^e^ Institute of Safety, Compensation & Recovery Research, Melbourne, Australia; ^f^ Caulfield Pain Management & Research Centre, Caulfield Hospital, Caulfield, Australia; ^g^ Department of Epidemiology & Preventive Medicine, Monash University, Melbourne, Australia; ^h^ College of Science and Technology, Temple University, Philadelphia, PA, USA; ^i^ Impact, National Knowledge and Advice Centre for Psychosocial Care Concerning Critical Incidents, Partner in Arq Psychotrauma Expert Group, Diemen, The Netherlands; ^j^ Department of Clinical and Health Psychology, Utrecht University, Utrecht, The Netherlands; ^k^ Division of Child and Adolescent Health Psychology, Department of Psychology, University of Zurich, Zurich, Switzerland; ^l^ Center for Injury Research and Prevention, Children’s Hospital of Philadelphia, Philadelphia, PA, USA; ^m^ School of Medicine, University of Pennsylvania Perelman, Philadelphia, PA, USA

**Keywords:** D-E-F protocol, emergency care, paramedics, paediatric injury, medical traumatic stress toolkit, PFA, Psychological First Aid, PTSD, traumatic stress

## Abstract

**Background:** Pre-hospital providers, such as paramedics and emergency medical technicians, are in a position to provide key emotional support to injured children and their families.

**Objective:** Our goal was to examine (a) pre-hospital providers’ knowledge of traumatic stress in children, attitudes towards psychosocial aspects of care, and confidence in providing psychosocial care, (b) variations in knowledge, attitudes, and confidence according to demographic and professional characteristics, and (c) training preferences of pre-hospital providers regarding psychosocial care to support paediatric patients and their families.

**Method:** We conducted a cross-sectional, online survey among an international sample of 812 pre-hospital providers from high-income countries. The questionnaire was adapted from a measure for a similar study among Emergency Department staff, and involved 62 items in 7 main categories (e.g. personal and work characteristics, knowledge of paediatric traumatic stress, and confidence regarding 18 elements of psychosocial care). The main analyses comprised descriptive statistics and multiple regression analyses.

**Results:** On average, respondents answered 2.7 (*SD *= 1.59) out of seven knowledge questions correctly. Respondents with higher knowledge scores were more often female, parent of a child under 17, and reported that at least 10% of their patients were children. A majority of participants (83.5%) saw all 18 aspects of psychosocial care as part of their job. Providers felt moderately confident (*M* = 3.2, *SD* = 0.45) regarding their skills in psychosocial care, which was predicted by gender (female), having more experience, having a larger proportion of child patients, and having received training in psychosocial care in the past five years. Most respondents (89.7%) wanted to gain more knowledge and skills regarding psychosocial care for injured children. In terms of training format, they preferred an interactive website or a one-off group training.

**Conclusions:** There appears to be both a need and an opportunity for education initiatives regarding paediatric traumatic stress in the pre-hospital context.

*‘If there is any time that you want to do everything absolutely right, it’s when you have a small child’* (Ambulance Nurse in Nordén, Hult, & Engström, 2014, p. 77). ‘*Kids are so scary. I mean, if you screw it up … you can’t imagine the repercussions’* (Emergency Medical Service provider in Cottrell et al., 2014, p. 355).

Caring for injured children is a stressful experience for paramedics and other ambulance staff. For many, there is minimal training in paediatric aspects of medical care, and typically no more than 10% of their patients are children, limiting opportunities to build up experience (Hansen et al., 2015; Remick, Caffrey, & Adelgais, 2014). Several interview studies report that paramedics and other pre-hospital providers experience high levels of stress when they receive a child trauma call (Avraham, Goldblatt, & Yafe, 2014; Gunnarsson & Stomberg, 2009; Nordén et al., 2014; Öberg, Vicente, & Wahlberg, 2015): it is a ‘very high risk, very low frequency’ event (Cottrell et al., 2014, p. 356). Moreover, providers rated this anxiety as highly likely to contribute to adverse patient events (Hansen et al., 2015).

At the same time, we know that injury is a potentially traumatic event for children and that medical providers can provide an important role in children’s experience (Horowitz, Kassam-Adams, & Bergstein, 2001). In a study of children injured in a traffic crash, over 80% developed at least one symptom of acute stress (e.g. efforts to avoid reminders, increased arousal; Winston et al., 2002). A minority of injured children develop persistent stress symptoms that can hinder their functioning and development in the long term (Alisic et al., 2014; Connor, Ford, Arnsten, & Greene, 2015). Similarly, parents are often affected; 83% of them reported at least one clinically significant symptom of acute stress in the immediate aftermath of a traffic crash (Winston et al., 2002), and a minority develop long-term stress symptoms (e.g. Kassam-Adams, Fleisher, & Winston, 2009).

Children’s long-term outcomes appear related to their initial experiences of threat and distress, as well as to their experiences of support (Alisic, Jongmans, Van Wesel, & Kleber, 2011; Marsac, Kassam-Adams, Delahanty, Widaman, & Barakat, 2014; Trickey, Siddaway, Meiser-Stedman, Serpell, & Field, 2012). Recently, several guidelines have been developed to alleviate survivors’ distress and increase self-efficacy after potentially traumatic events. Psychological First Aid (PFA; Brymer et al., 2006) is a prominent model frequently applied after disasters. PFA comprises eight elements, which are used according to the needs of the survivor: (1) contact and engagement, (2) ensuring safety and comfort, (3) stabilization (e.g. calming), (4) gathering information regarding current needs and concerns, (5) practical assistance, (6) promoting connection with social supports, (7) informing about coping, and (8) linking with collaborative services. International guidelines have recommended the use of PFA principles in the immediate aftermath of disaster and other trauma (Forbes et al., 2010). While PFA’s focus is on the post-disaster context, the D-E-F protocol (Stuber, Schneider, Kassam-Adams, Kazak, & Saxe, 2006) provides specific recommendations for the paediatric context (see also Kassam-Adams, 2014). It builds on the A-B-C model (airway, breathing, and circulation), which is familiar to acute care clinicians providing resuscitation. After the ABC’s and other physical health needs have been addressed, the protocol points providers to address the distress of the patient (D), provide emotional support for the patient (E), and consider the family (F) (Kassam-Adams, Marsac, Hildenbrand, & Winston, 2013). Both the PFA and D-E-F models may help assess and guide trauma-informed care by emergency care providers, including pre-hospital providers (see also Magruder, Kassam-Adams, Thoresen, & Olff, 2016).

The goal of the present study was to understand pre-hospital providers’ perspectives on psychosocial elements of care for injured children. In particular, we aimed to examine (a) pre-hospital providers’ knowledge of traumatic stress in children, attitudes towards psychosocial aspects of care, and confidence in providing psychosocial care, (b) to what extent variations in knowledge, attitudes, and confidence vary according to demographic and professional characteristics, and (c) what training preferences pre-hospital providers have when it comes to enhancing knowledge and confidence of psychosocial care to support paediatric patients and their families.

## 1. Method

We conducted a cross-sectional, online survey among an international sample of paramedics and other pre-hospital providers from high-income countries. The Human Research Ethics Committee of Monash University approved the study (#CF14/1167–2014000519).

### 1.1. Participants

We recruited participants via paramedic and ambulance organizations, unions, university departments of emergency care and relevant professional associations in the USA, Canada, Australia, New Zealand, Switzerland, Austria, and the UK. Respondents were eligible if they were currently working as a pre-hospital provider and for this article we selected only those who were active in the countries mentioned. Data collection took place from June 2014 until November 2014. To reduce any barriers to providing a frank account of organizational performance, participation in the survey was anonymous. Participants indicated consent by completing the questionnaire. They were also asked to forward the questionnaire to colleagues in their network.

### 1.2. Materials

We tailored the measure from one in a similar study among hospital Emergency Department (ED) staff (Alisic et al., 2016) so that it was appropriate for pre-hospital providers. Modifications were informed by a review of the literature regarding pre-hospital care and through consultation and pilot testing with providers, ambulance management staff, and topic experts. We removed questions that were not relevant (e.g. regarding working in an academic versus non-academic hospital) and added a few others instead (e.g. participants’ parental status), based on providers’ and experts’ feedback.

The questionnaire was available in English through the online platform SurveyMonkey, and the part relevant to this article consisted of 62 items in seven main categories: personal and work characteristics (demographics, profession and work location; 10 items); individual knowledge of traumatic stress (seven multiple choice items); individual confidence in, and attitudes towards providing psychosocial care (mapped on the eight core elements of PFA; 18 items with a 4-point Likert scale ranging from 1 to 4 and an option to indicate that the provider thought it was ‘not part of my job’); barriers to providing psychosocial care (six items with a 3-point Likert scale ranging from 1 to 3, and one open question); their ambulance service’s performance in providing psychosocial care (three general questions and eight items for each element of PFA, all with a 4-point Likert scale ranging from 1 to 4 and the ‘not part of our job’ option); training wishes and training experiences with regard to psychosocial care for injured children (seven items with varying answer formats); and further comments (two open questions). The questionnaire is available as a Supplementary File.

### 1.3. Data analysis

We conducted all analyses in IBM SPSS version 22. We derived a total knowledge score as a count of correctly answered knowledge questions (0–7). A total attitude score comprised the count of psychosocial care elements (0–18) seen as part of the respondent’s job. For each of the elements, we computed an average confidence score only among those who saw it as part of their job. We computed a total average confidence score for all participants who saw at least 12 of the 18 aspects as part of their job (99% of the sample). We used descriptive statistics to give an overview of the respondents’ perspectives and non-hierarchical multiple linear regression analyses to examine which respondent characteristics (i.e. age, gender, parental status, profession, experience, proportion of child patients, and recent training in psychosocial care) related to higher knowledge and confidence scores. We dichotomized gender (male vs. female; leaving out ‘prefer not to say’) and profession (paramedics vs. emergency medical technicians, leaving out ‘other’; in both cases the third category involved only a few participants). We report the initial models with all potential predictors as well as the final models that include significant factors only (cf. Field, 2009). Because age and years of experience in patient care were strongly correlated (*r* = .76; *p* < .001), we included only years of experience in patient care in the regression models.

## 2. Results

### 2.1. Respondents

The sample consisted of 812 pre-hospital providers (31.8% female, 67.7% male, and 0.5% preferred not to say) with a mean age of 39.3 years (range 18–65; *SD *= 10.6; *Mdn* = 39.0). Most respondents worked in Canada (32.1%), followed by the USA (27.5%), Australia (19.3%), and New Zealand (9.5%). The majority worked in a publicly owned ambulance service (70.6%) as opposed to a privately owned service (20.8%; 8.6% were unsure or preferred not to say). Participants were fairly evenly distributed across settings: 35.1% worked in mostly suburban areas, while 35% served mostly rural areas and 29.9% practiced in a mostly urban or inner city setting. Almost half of the sample (45.3%) had children under the age of 17. The majority of the respondents self-identified as (senior) paramedics (77.5%) or (advanced) emergency medical technicians (EMT; 18.9%), with the remaining 3.6% identifying as emergency medical responders, emergency care assistants or trainees. On average, the respondents had 13.8 years of experience as a pre-hospital provider (*Mdn *= 12.0, *SD = *9.4). With regard to the patients they served, most saw relatively few children (aged 0–16 years) compared to adults: about a third (36.3%) estimated that less than 5% of their primary patients were children, while 42.1% reported that 5–10% of their patients were children, and 17.4% estimated the percentage to be 10–20%.

### 2.2. Knowledge of paediatric traumatic stress

On average, respondents answered 2.7 (*SD* = 1.6) out of seven questions correctly (see Table 1). Participants were most aware of the fact that all family members were at risk of developing stress symptoms and least aware of the large proportion of children who can experience posttraumatic stress after injury. Regarding age groups at risk of posttraumatic stress, there was only limited recognition (by 32.5% of the respondents) that toddlers should be included as an age group that can develop stress symptoms. Considering presenting behaviours, 79.4% recognized that children who were quiet or withdrawn could go on to develop stress symptoms, but regarding loud, calm, or frantic behaviour, these figures were only 32.8%, 35.3%, and 51.6% respectively. The regression analyses showed that respondents with higher knowledge scores were more often female, parent of a child under 17, and reported that at least 10% of their patients were children. However, these characteristics explained only 5.8% of the variance in knowledge scores (see Table 2). Profession (paramedic versus EMT), years of experience in patient care, and having received training in psychosocial care for children in the past five years were not significantly associated with knowledge scores.

**Table 1.** Pre-hospital providers’ knowledge of traumatic stress in children.

Knowledge item	*N* (%) responding correctly
All injury severities are at risk for traumatic stress	386 (47.5)
All age groups are at risk for traumatic stress	223 (27.5)
The child, parents, and siblings are at risk for traumatic stress	625 (77.0)
Various behaviours (e.g. calm, frantic) can precede traumatic stress	197 (24.3)
Subjective life threat is a risk factor	474 (58.4)
Pain experience is a risk factor	268 (33.0)
> 50% of children report stress symptoms in 1st month post-injury	21 (2.6)

*N *= 812.

**Table 2.** Respondents’ total knowledge score in relation to their characteristics: initial and final multiple regression.

Initial model	*B*	*SE B*	*β*	*p* value	95% CI for *B*	Univariate total scores per group/correlations^a^			
Constant	2.086	.136		<.001	1.819 to 2.354	*Coded ‘0’*	*M (SD)/r*	*Coded ‘1’*	*M (SD)*
Gender	.748	.123	.221	<.001	.507 to .989	Male	2.50 (1.56)	Female	3.16 (1.54)
Parent	.267	.112	.085	.017	.047 to .487	No	2.64 (1.59)	Yes	2.78 (1.58)
Profession	−.103	.138	−.026	.457	−.374 to .169	Paramedic	2.72 (1.57)	EMT	2.62 (1.62)
Experience (in years)^b^	.013	.006	.077	.033	.001 to .025		.011		
Child patients	.361	.135	.094	.008	.096 to .626	< 10%	2.64 (1.58)	≥ 10%	2.93 (1.59)
Recent training	.372	.213	.061	.082	−.047 to .790	No	2.67 (1.58)	Yes	3.14 (1.56)
Final model	*B*	*SE B*	*β*	*P* Value	95% CI for *B*				
Constant	2.302	.091		<.001	2.123 to 2.481				
Gender	.712	.118	.210	<.001	.480 to .945				
Parent	.248	.111	.078	.026	.030 to .465				
Child patients	.314	.133	.081	.018	.053 to .574				

*N *= 780 for the initial model and 808 for the final model; these sample sizes differ due to a greater degree of missing data for ‘Profession’. ‘Profession’ distinguishes between (senior) paramedics and (advanced) Emergency Medical Technicians. ‘Child patients’ refers to the proportion of children among the participants’ patients (< 10% vs. ≥ 10%). ‘Recent training’ refers to training in psychosocial care for injured children in the past five years. ^a^For the univariate descriptives, we used all information available; *N* was 812 for all variables, except for ‘Gender’ (808) and ‘Profession’ (784). ^b^No longer significant when ‘Profession’ and ‘Recent training’ were removed from the model. Adjusted *R^2^* of the final model = .05, *F*(3,804)* *= 14.48, *p *< .001.

### 2.3. Views on psychosocial care

A majority of participants (83.5%) saw all 18 aspects of psychosocial care as part of their job, and each aspect was viewed as part of the job by over 90% of the respondents. Among those aspects that were sometimes seen as *not* part of one’s job, the most frequent were: teaching coping skills during medical procedures, educating parents about signs of a child’s need for mental health support in the future, and educating families about how to access this support (see Table 3). Because of the lack of variance in the total score (96.2% saw at least 14 aspects of psychosocial care as part of their job), we did not further analyse predictors of to what extent respondents felt psychosocial care to be part of their role.

**Table 3.** Elements of psychosocial care perceived as part of the job.

Aspect of psychosocial care	‘not my job’ *N* (%)
Respond calmly and without judgment to a child’s or family’s strong emotional distress	9 (1.1)
Talk with children in age appropriate language	8 (1.0)
Tailor your approach according to a family’s cultural background	10 (1.2)
Assess and manage pain in children	9 (1.1)
Explain procedures to children and parents	8 (1.0)
Inform a child about an injured/deceased family member	22 (2.7)
Help a child/parent who is anxious to calm down by teaching relaxation (e.g. breathing) techniques	11 (1.4)
Assess a child’s or family’s distress, emotional needs, and support systems	13 (1.6)
Elicit trauma details from a child or family without them being exposed to more distress	8 (1.0)
Respond to a child’s (or parent’s) question about whether the child will die	14 (1.7)
Liaise with staff who can provide practical assistance to a family (e.g. Social Work)	29 (3.6)
Take action to get someone close (a parent, family member or friend) available to the child	11 (1.4)
Encourage parents to make use of their own social support system (family, friends, spiritual community, etc.)	21 (2.6)
Educate children and families about common traumatic stress reactions	43 (5.3)
Teach parents or children specific ways to cope with procedures	64 (7.9)
Provide information to parents about emotional or behavioural reactions that indicate that the child may need help (when back at home)	75 (9.2)
Educate parents or children about how to access mental health services if needed	55 (6.8)
Manage your own emotional responses to children’s pain and trauma	7 (0.9)

*N *= 812. The three aspects of psychosocial care that had the highest percentages, are highlighted.

### 2.4. Confidence in psychosocial care skills

On average, pre-hospital providers felt moderately confident (*M* = 3.2, *SD* = 0.45) regarding psychosocial care. They reported varying levels of confidence regarding different aspects of psychosocial care. For example, they felt most confident about explaining procedures to children and parents and least confident about informing parents about signs of need for further mental health care (see Table 4). The regression analyses showed that a higher level of confidence was associated with being female, having more experience, having a larger proportion of child patients, and having received training in psychosocial care for injured children in the past five years. These characteristics explained 4.4% of the variance in average confidence scores (see Table 5). Parental status and profession (paramedic versus EMT) were not significantly associated with confidence in providing psychosocial care. Respondents’ confidence in their own psychosocial care performance (*M *= 3.2; *SD* = 0.45) was significantly higher than their assessment of the performance of their ambulance service as a whole (*M* = 2.1; *SD* = .89; paired samples *t*-test: *t *= 35.0, *df *= 730; *p* < .001; please note the different stems of the Likert scales, see Supplemental File). While confusing evidence and worries about upsetting children and families were seen as significant barriers by a minority of the respondents (16.1% and 12.7% respectively), more participants were concerned about time constraints (34.0%), lack of dedicated space to provide psychosocial care (32.8%), lack of support from supervisors (33.3%), and especially the lack of training (44.6%).

**Table 4.** Respondents’ level of confidence regarding aspects of psychosocial care.

Aspect of psychosocial care	Mean score^a^ (SD)
Respond calmly and without judgment to a child’s or family’s strong emotional distress	3.70 (0.53)
Talk with children in age appropriate language	3.66 (0.56)
Tailor your approach according to a family’s cultural background	3.19 (0.69)
Assess and manage pain in children	3.40 (0.69)
Explain procedures to children and parents	3.78 (0.46)
Inform a child about an injured/deceased family member	2.92 (0.87)
Help a child/parent who is anxious to calm down by teaching relaxation (e.g. breathing) techniques	3.37 (0.71)
Assess a child’s or family’s distress, emotional needs, and support systems	3.21 (0.73)
Elicit trauma details from a child or family without them being exposed to more distress	2.98 (0.77)
Respond to a child’s (or parent’s) question about whether the child will die	3.09 (0.79)
Liaise with staff who can provide practical assistance to a family (e.g. Social Work)	3.24 (0.84)
Take action to get someone close (a parent, family member or friend) available to the child	3.53 (0.67)
Encourage parents to make use of their own social support system (family, friends, spiritual community, etc.)	3.37 (0.72)
Educate children and families about common traumatic stress reactions	2.71 (0.88)
Teach parents or children specific ways to cope with procedures	2.66 (0.88)
Provide information to parents about emotional or behavioural reactions that indicate that the child may need help (when back at home)	2.55 (0.94)
Educate parents or children about how to access mental health services if needed	2.80 (0.91)
Manage your own emotional responses to children’s pain and trauma	3.28 (0.74)

*N *= 737–805. The three aspects of psychosocial care that had the lowest mean scores, are highlighted. ^a^Answer options to indicate confidence regarding each element of psychosocial care were (1) not at all; (2) a little; (3) moderately; (4) very.

**Table 5.** Respondents’ average confidence score in relation to their characteristics: initial and final multiple regression.

Initial model	*B*	*SE B*	*β*	*p* value	95% CI for *B*	Univariate total scores per group/correlations^a^			
Constant	3.016	.039		<.001	2.939 to 3.093	*Coded ‘0’*	*M (SD)/r*	*Coded ‘1’*	*M (SD)*
Gender	.106	.035	.110	.003	.037 to .176	Male	3.18 (0.45)	Female	3.25 (0.45)
Parent	.057	.032	.064	.076	−.006 to .121	No	3.18 (0.47)	Yes	3.23 (0.43)
Profession	.036	.040	.032	.364	−.042 to .114	Paramedic	3.19 (0.44)	EMT	3.22 (0.48)
Experience (in years)^b^	.005	.002	.112	.002	.002 to .009		.096		
Child patients	.125	.039	.113	.001	.048 to .201	< 10%	3.18 (0.47)	≥ 10%	3.29 (0.40)
Recent training	.223	.062	.127	<.001	.102 to .344	No	3.18 (0.45)	Yes	3.43 (0.41)
Final model	*B*	*SE B*	*β*	*p* value	95% CI for *B*				
Constant	3.047	.034		<.001	2.980 to 3.113				
Gender	.103	.034	.107	.003	.036 to .171				
Experience (in years)	.006	.002	.119	.001	.002 to .009				
Child patients	.122	.038	.111	.001	.047 to .197				
Recent training	.229	.061	.130	<.001	.109 to .348				

*N *= 774 for the initial model and 801 for the final model. ‘Profession’ distinguishes between (senior) paramedics and (advanced) Emergency Medical Technicians. ‘Child patients’ refers to the proportion of children among the participants’ patients (< 10% vs. ≥ 10%). ‘Recent training’ refers to training in psychosocial care for injured children in the past five years. ^a^For the univariate descriptives, we used all information available; *N* was 805 for all variables, except for ‘Gender’ (801) and ‘Profession’ (778). Adjusted *R^2^* of the final model = .044, *F*(4,796)* *= 10.20, *p *< .001.

### 2.5. Training needs and preferences

Only 7.1% of the respondents had received training in psychosocial care for children in the past five years. The training model referred to most often was Critical Incident Stress Debriefing (Mitchell & Everly, 1996; currently not recommended for children; Foa, Keane, Friedman, & Cohen, 2009). Most respondents (89.7%) wanted to gain more knowledge and skills regarding psychosocial care for injured children. A small number of participants felt that they had sufficient skills already (4.2%) or that it was not relevant for them (4.2%). For 2.0% there were other reasons, such as wanting to leave the profession or personal issues. For those who wanted training, the two most popular training modes were an interactive website (24.6% of first preferences) and one-off group training (20.7% of first preferences; Table 6). In addition, respondents made several suggestions, e.g. to distribute articles with evidence-based recommendations and to place a stronger emphasis on the topic in initial paramedic education. Of those interested in training, 35.4% indicated they would be able to commit 1–4 hours to it in the next six months, 31.6% could commit 5–8 hours, and 33.0% could commit more than eight hours.

**Table 6.** Respondents’ preferences regarding training format.

	1^st^ preference *N* (%)	2^nd^ preference *N* (%)
Book	49 (6.0)	84 (10.3)
Static website	71 (8.7)	97 (11.9)
Interactive website	200 (24.6)	129 (15.9)
Mentoring by paramedic	66 (8.1)	66 (8.1)
Mentoring by MH clinician	89 (11.0)	74 (9.1)
One-off group training	168 (20.7)	135 (16.6)
Multi-session group training	82 (10.1)	114 (14.0)

*N *= 728 respondents interested in training regarding psychosocial care. MH = mental health.

## 3. Discussion

Most pre-hospital providers in our international survey saw psychosocial aspects of care as important and part of their role, and reported that they were moderately confident about applying psychosocial skills in the care of injured children. However, we identified a number of gaps in knowledge of paediatric traumatic stress. While almost none of the respondents had received adequate training in psychosocial care, a large majority endorsed a desire for it. Demographic and professional factors only explained a small amount of the variance in providers’ knowledge and confidence scores, highlighting that knowledge, attitudes, and confidence in delivering psychosocial care are broadly appreciated but not strongly attributable to these individual worker characteristics.

In terms of pre-hospital providers’ knowledge, gains can be made regarding awareness of the diversity and number of children who can develop traumatic stress symptoms. This is in line with a previous study with ED staff (Alisic et al., 2016), and has implications for clinical training and practice. For example, if pre-hospital providers assume that only children with a quiet or withdrawn presentation are at risk for traumatic stress, they may discount the need to provide effective support for children who have a different behavioural and affective presentation. The same applies to developmental levels; young children may be overlooked as at risk of traumatic stress symptoms. Nevertheless, pre-hospital providers’ ratings of the importance of psychosocial aspects of care were high, which is a key starting point for trauma-informed care (e.g. see Fraser et al., 2014). Similar to the findings in the ED study, the elements of psychosocial care that were most frequently viewed as ‘not part of the job’ were also aspects with low confidence ratings among those providers who did see them as part of the job. Although high, the overall ratings of the importance of psychosocial care appeared to be slightly lower in the pre-hospital providers’ sample than in the ED sample (Alisic et al., 2016), which may reflect the shorter duration of each clinical encounter for pre-hospital providers, possibly in combination with a focus on ‘load and go’ (Cottrell et al., 2014). Considering the frequently mentioned time constraints, it may be worthwhile to establish a hierarchy of PFA elements for pre-hospital providers. For example, stabilization through calming may be more urgent in the pre-hospital context than connecting with sources of social support. A Delphi study – involving both patients and providers – on prioritization of psychosocial care elements may be useful.

Interestingly, training experience did not significantly relate to knowledge and only to a minor extent to confidence. There are several potential explanations for this finding. First, only a small minority of providers reported previous training so there may have been a lack of variance due to training per se. Second, the training model that was most often reported was Critical Incident Stress Debriefing, which has been controversial and contra-indicated in recent years (although for children the evidence base is less clear; Jacobs & Pfefferbaum, 2015). Third, the training received most likely focused on general psychosocial care, including care for co-workers, and may not have specifically focused on paediatric stress. Nevertheless, participants expressed a clear need for and interest in further education on providing psychosocial care to their paediatric patients. Because infrequent exposure to paediatric patients in the field gives pre-hospital providers little chance to hone their skills in this area via on-the-job training, there appears to be a need for formal training opportunities. These could be made available as a Continuing Education module for practicing pre-hospital providers (e.g. via online programs, as indicated by the current respondents), or implemented as part of initial training and education. Training that involves realistic, high fidelity simulation of paediatric cases may hold particular promise in this respect, especially considering the many respondents interested in group-based training. Simulation allows for granular observation and feedback regarding new skills, and practice of skills that are used infrequently in a provider’s usual practice (Abelsson, Rystedt, Suserud, & Lindwall, 2014). Engaging paediatricians, specialists in the design and implementation of trauma-informed care, and experts in pre-hospital care will ensure that the training has a strong clinical and scientific evidence base.

The present study has a number of limitations. First, while it is an international survey, it includes only a selection of high-income countries, related to where we had access to networks of providers. The findings may not be generalizable to other high-income countries, and are unlikely to be generalizable to low- or middle-income countries. It is essential that more research be done in low- and middle-income countries, since resources are fewer and trauma-exposure is more prevalent (see e.g. Fodor et al., 2014; Schnyder, 2013). Second, while this survey provides insight regarding providers’ knowledge and perspectives, it is only an indirect measure of their actual behaviour and skills. In addition, the provider characteristics that we selected explained only a small part of the variance in knowledge and confidence. It is possible that factors such as the organizational culture in which providers operate play a more important role. Finally, there may have been a selection bias, with those more interested in psychosocial care more likely to participate in the study, although at least some respondents showed a critical view (e.g. one participant wrote: *‘I don’t care about those things; so typically I don’t bother. There are people that get paid to do that, I’m not one of them’*).

Relevant questions that remain for further research include whether receiving training in paediatric care enables providers to feel less stressed and improves the quality of care that paediatric patients receive (cf. Hansen et al., 2015). In addition, it appears relevant to better understand how children and parents experience their interactions with pre-hospital providers, and what role pre-hospital providers play in modifying (i.e. increasing or decreasing) physiological and psychological arousal in paediatric patients during the peri-trauma period. Currently, the guidelines for health care providers’ interactions with paediatric patients in emergency care are grounded in an empirical evidence base about risk factors for traumatic stress, and based on international expert consensus. However, there are no clear empirical data on their effectiveness in preventing the development of traumatic stress. Research that evaluates whether certain elements of psychosocial care, as delivered by pre-hospital providers, have a greater impact on child and family outcomes than others would be a valuable addition to the field. The combination of these findings with the Delphi study recommended above to generate a hierarchy of psychosocial care priorities may lead to a helpful prioritization for pre-hospital providers.

## 4. Conclusions

The current study shows a need and an opportunity for education initiatives regarding paediatric traumatic stress in the pre-hospital context. Collaborative efforts among providers, educators, patients, and their families may help improve care in situations that are stressful for both the children and the adults involved.

## Supplementary Material

Supplementary material
