# Supplementary material for: Trauma-informed care for children in the ambulance: international survey among pre-hospital providers
Source: Eur J Psychotraumatol. 2017 Feb 8;8(1):1273587. doi: 10.1080/20008198.2016.1273587 (PMC5328382; doi:10.1080/20008198.2016.1273587)
Supplement: Supplementary material [file zept_a_1273587_sm0241.pdf]

Please tell us about yourself:

1. What is your gender?

- ◯ Female
- ◯ Male
- ◯ Prefer not to say

2. What is your age?

[ ]

3. Do you have children under the age of 17?

- ◯ Yes
- ◯ No

4. Are you currently training or working as a paramedic or other emergency health service provider (including graduate paramedic and ambulance driver)?

- ◯ Yes, working as a paramedic
- ◯ Yes, working as an emergency medical responder
- ◯ Yes, working as an emergency medical technician
- ◯ Yes, working as an advanced emergency medical technician
- ◯ Yes, training/studying to be a paramedic/emergency medical service provider
- ◯ No, I am on leave
- ◯ No, I no longer work as a paramedic (this questionnaire is not applicable for you)

5. How many years of experience do you have working as a paramedic or other emergency health service provider?

[ ]

6. How many of your primary patients are children (0-16 years)?

- ◯ less than 5%
- ◯ 5 – 10%
- ◯ 10 – 20%
- ◯ 20 – 40%
- ◯ 40 – 60%
- ◯ more than 60%

7. Is the ambulance service you work for publicly or privately owned?

- ◯ Publicly owned
- ◯ Privately owned
- ◯ I'm not sure

Other (please specify)

[ ]

8. What location do you work in?

- ◯ mostly rural area
- ◯ mostly suburban area
- ◯ mostly urban/inner city area

Other (please specify)

[ ]

9. Which country do you work in?

10. Which state, province or region do you work in?

Based on your experience and knowledge, please answer the following:

Please keep in mind:
For some questions you may choose multiple answers. Please tick - or check - all that apply.
PTS = children's long-term posttraumatic stress reactions, for example nightmares and concentration difficulties a month after the injury.

11. What severity of injury puts children at risk of Post Traumatic Stress (PTS)? Please tick all that apply:

- [ ] Minor injury (e.g., superficial laceration, dental injury)
- [ ] Moderate injury (e.g., closed limb fracture, facial fracture)
- [ ] Serious injury (e.g., open limb fracture, hemothorax)
- [ ] Severe or critical injury (e.g., ruptured liver, proximal limb amputation)
- [ ] I'm not sure

12. Which age groups are at risk of PTS following an injury? Please tick all that apply:

- [ ] Toddlers (1 to 3 years)
- [ ] Young children (4 to 7 years)
- [ ] Older children (8 to 12 years)
- [ ] Adolescents (13 to 18 years)
- [ ] I'm not sure

13. Who is at risk of PTS following the injury of a child? Please tick all that apply:

- [ ] The injured child
- [ ] Parents
- [ ] Siblings
- [ ] I'm not sure

14. Children at risk of PTS present as... Please tick all that apply:

- [ ] Frantic and distressed
- [ ] Loud
- [ ] Calm and compliant
- [ ] Quiet / withdrawn
- [ ] I'm not sure

15. Children who, at some point during the trauma, believe that they might die are at higher risk of PTS.

- ◯ Agree
- ◯ Agree, but only when that belief is based on a realistic probability of death
- ◯ Disagree
- ◯ I'm not sure

16. Injured children who rate their pain as severe...

- ◯ Have the same risk of PTS as other children
- ◯ Are at increased risk of PTS
- ◯ Are at lower risk of PTS
- ◯ I'm not sure

17. What percentage of injured children and families report traumatic stress reactions within the first month after the injury?

- ◯ less than 25%
- ◯ 25% - 50%
- ◯ more than 50%
- ◯ I'm not sure

18. How confident are you as a provider that you can...

	Not at all	A little	Moderately	Very	This is not part of my job
Respond calmly and without judgment to a child's or family's strong emotional distress	◯	◯	◯	◯	◯
Talk with children in age appropriate language	◯	◯	◯	◯	◯
Tailor your approach according to a family's cultural background	◯	◯	◯	◯	◯
Assess and manage pain in children	◯	◯	◯	◯	◯
Explain procedures to children and parents	◯	◯	◯	◯	◯
Inform a child about an injured / deceased family member	◯	◯	◯	◯	◯

19. How confident are you as a provider that you can...

	Not at all	A little	Moderately	Very	This is not part of my job
Help a child / parent who is anxious to calm down by teaching relaxation (e.g. breathing) techniques	◯	◯	◯	◯	◯
Assess a child's or family's distress, emotional needs, and support systems	◯	◯	◯	◯	◯
Elicit trauma details from a child or family without them being exposed to more distress	◯	◯	◯	◯	◯
Respond to a child's (or parent's) question about whether the child will die	◯	◯	◯	◯	◯
Liaise with staff who can provide practical assistance to a family (e.g. Social Work)	◯	◯	◯	◯	◯
Take action to get someone close (a parent, family member or friend) available to the child	◯	◯	◯	◯	◯

20. How confident are you as a provider that you can...

	Not at all	A little	Moderately	Very	This is not part of my job
Encourage parents to make use of their own social support system (family, friends, spiritual community, etc.)	◯	◯	◯	◯	◯
Educate children and families about common traumatic stress reactions	◯	◯	◯	◯	◯
Teach parents or children specific ways to cope with procedures	◯	◯	◯	◯	◯
Provide information to parents about emotional or behavioural reactions that indicate that the child may need help (when back at home)	◯	◯	◯	◯	◯
Educate parents or children about how to access mental health services if needed	◯	◯	◯	◯	◯
Manage your own emotional responses to children's pain and trauma	◯	◯	◯	◯	◯

The term 'psychosocial care' refers to the various actions described in the questions above.

21. Please indicate whether any of the following are barriers for you in providing psychosocial care

	Not a barrier	Somewhat a barrier	Significant barrier
Time constraints	○	○	○
Lack of training	○	○	○
Confusing evidence on what to do	○	○	○
Worry about further upsetting children and families	○	○	○
Lack of a dedicated space to provide psychosocial care	○	○	○
Lack of support from supervisors / others in the system that you work in	○	○	○

22. Are there any other barriers for you in providing psychosocial care? Please explain

23. How important is it that paramedics or or other emergency health service providers help children and families cope with traumatic stress reactions (e.g. by providing psychosocial care) in your view?

- ○ Not important
- ○ Somewhat important
- ○ Very important

Other (please specify)

24. Please rate the performance of your workplace as a whole on each of the following:

	Poor	Fair	Good	Excellent	This is not part of our job
Providing psychosocial care to injured children and their families	○	○	○	○	○
Helping staff manage their own emotional responses to patients' pain and trauma	○	○	○	○	○
Using scientific evidence as a basis for psychosocial care for patients and staff	○	○	○	○	○

25. What is the performance of your workplace as a whole on the following aspects of psychosocial care for injured children and their families?

	Poor	Fair	Good	Excellent	This is not part of our job
1. **Contact and engagement** (responding to or initiating contacts in a non-intrusive, compassionate, and helpful manner)	◯	◯	◯	◯	◯
2. **Safety and comfort** (enhancing immediate and ongoing safety, and provide physical and emotional comfort)	◯	◯	◯	◯	◯
3. **Stabilization** (calming and orienting emotionally overwhelmed/distraught children/families)	◯	◯	◯	◯	◯
4. **Information gathering on current needs & concerns**	◯	◯	◯	◯	◯
5. **Practical assistance** (offering practical help to children/families in addressing immediate needs and concerns)	◯	◯	◯	◯	◯
6. **Connecting children/families with social supports**	◯	◯	◯	◯	◯
7. **Giving information on coping**	◯	◯	◯	◯	◯
8. **Linking children/families with collaborative services**	◯	◯	◯	◯	◯

The following questions relate to psychological support for paramedics or or other emergency health service providers in your workplace.

26. Are there trained peer-support officers in your ambulance service?

◯ Yes

◯ No

◯ I don't know

27. Have you received any professional support following a critical incident (e.g. through a peer-support program)?

◯ Yes

◯ No

28. If you answered yes to question 27, for approximately how many incidents have you received support?

◯ 1 – 3

◯ 4 – 7

◯ More than 7

29. If you answered yes to question 27, how helpful was the support you received?

- ◯ Not helpful
- ◯ Somewhat helpful
- ◯ Very helpful

30. To what extent are these statements true in your workplace?

	Strongly disagree	Disagree	Agree	Strongly agree
People I work with are competent in doing their jobs	◯	◯	◯	◯
People I work with take a personal interest in my wellbeing in the face of traumatic incidents at work	◯	◯	◯	◯
People I work with are friendly	◯	◯	◯	◯
People I work with are helpful in supporting colleagues after a distressing incident	◯	◯	◯	◯
Management is concerned about the wellbeing of those under them in the face of traumatic incidents at work	◯	◯	◯	◯
Management pays attention to what you are saying with regard to staff wellbeing	◯	◯	◯	◯
Management is helpful in supporting staff after a distressing incident	◯	◯	◯	◯
Management is successful in getting people to work together	◯	◯	◯	◯
The ambulance organisation is helpful in supporting staff after a distressing incident	◯	◯	◯	◯
The ambulance organisation is concerned about the wellbeing of staff in the face of traumatic incidents at work	◯	◯	◯	◯
I have enough time to recover from distressing incidents at work.	◯	◯	◯	◯

The following questions relate to your general levels of psychological distress and wellbeing.

31. During the last 30 days, about how often did you feel...

	None of the time	A little of the time	Some of the time	Most of the time	All of the time
Depressed?	◯	◯	◯	◯	◯
So depressed that nothing could cheer you up?	◯	◯	◯	◯	◯
Hopeless?	◯	◯	◯	◯	◯
Restless or fidgety?	◯	◯	◯	◯	◯
So restless that you could not sit still?	◯	◯	◯	◯	◯

32. During the last 30 days, about how often did you feel...

	None of the time	A little of the time	Some of the time	Most of the time	All of the time
Tired out for no good reason?	◯	◯	◯	◯	◯
That everything was an effort?	◯	◯	◯	◯	◯
Worthless?	◯	◯	◯	◯	◯
Nervous?	◯	◯	◯	◯	◯
So nervous that nothing could calm you down?	◯	◯	◯	◯	◯

The following questions are related to training in psychosocial care.

33. Would you like to gain more knowledge and skills regarding psychosocial care for injured children?

- ◯ Yes
- ◯ No, my knowledge and skills are sufficient
- ◯ No, I feel it is not relevant
- ◯ No, because

34. If you have answered yes to question 33, which training format would you prefer? Please indicate your 1st and 2nd choice

	1st preference	2nd preference
A book on the topic	◯	◯
Online: website with written information	◯	◯
Online: interactive website (e.g., 'webinar', video examples, quizzes)	◯	◯
Individual mentor sessions with an experienced clinician of my own profession	◯	◯
Individual mentor sessions with a mental health clinician	◯	◯
Group training in-person in one block of hours	◯	◯
Group training in-person spread over a number of weeks	◯	◯

Other (please specify)

35. If you have answered yes to question 33, how many hours of training would you be able to commit to (in the next 6 months)?

- ◯ 1 – 4 hrs
- ◯ 5 – 8 hrs
- ◯ > 8 hrs

36. Have you had specific training in psychosocial care for injured children?

- ◯ No
- ◯ Yes

37. If you have answered yes to question 36, how many hours of training did you have?

- ◯ 1 – 4 hrs
- ◯ 5 – 8 hrs
- ◯ > 8 hrs

38. If you have answered yes to question 36, how long ago was the training?

- ◯ within the past year
- ◯ 1 to 5 years ago
- ◯ 5 to 10 years ago
- ◯ more than 10 years ago

39. If you have answered yes to question 36, what was the model/method used?

- ◯ MANERS
- ◯ Psychological First Aid
- ◯ Medical Traumatic Stress Toolkit
- ◯ Critical Incident Stress Debriefing
- ◯ Other - I don't know the name any more
- ◯ Other - the model/method was:

40. We involve paramedics and other emergency health service providers from as many countries as possible. Are there any cultural considerations/issues that you would like to make us aware of? Please share them here:

41. If you have any additional comments on working with injured children and families, please share them here:

Many thanks for your participation.

Thank you!
